# Characterization of Histopathological and Ultrastructural Changes in Channel Catfish Experimentally Infected with Virulent *Aeromonas hydrophila*

**DOI:** 10.3389/fmicb.2017.01519

**Published:** 2017-08-15

**Authors:** Hossam Abdelhamed, Iman Ibrahim, Wes Baumgartner, Mark L. Lawrence, Attila Karsi

**Affiliations:** ^1^Department of Basic Sciences, College of Veterinary Medicine, Mississippi State University, Starkville MS, United States; ^2^Department of Pathology, Faculty of Veterinary Medicine, Mansoura University Mansoura, Egypt; ^3^Department of Pathobiology and Population Medicine, College of Veterinary Medicine, Mississippi State University, Starkville MS, United States

**Keywords:** vAh, histopathology, electron microscopy, eosinophilic granular cells, DC-like cells

## Abstract

A highly virulent clonal population of *Aeromonas hydrophila* (vAh) has been the cause of recent motile *Aeromonas* septicemia epizootic in channel catfish (*Ictalurus punctatus*) farms in the Southeastern United States. The pathology of the disease caused by vAh has not been studied well yet. Thus, our aim was to determine histopathological and ultrastructural changes in channel catfish following vAh challenge. To accomplish this, catfish fingerlings were challenged with vAh (strain ML09-119) by bath. Six fish per each time point were collected at 1, 3, 5, 6, 24, and 48 h for light microscopy, and six fish were collected at 48 h for transmission electron microscopy (TEM). The first pathological lesions were detected in the spleen and stomach at 1 h post-challenge (HPC) while intestine, gills, kidney, and liver lesions were observed at 24 and 48 HPC. Histopathological examination revealed degenerative changes, necrosis, extensive edema, and inflammation in internal organs. The TEM showed severe tissue destruction with multiple bacterial cells secreting outer membrane vesicles, especially in spleen and gills and far number in the stomach. Degenerated bacterial cells were observed in the intestinal lumen and the phagosomes of phagocytic kidney cells. We identified, for the first time, degranulate eosinophilic granular cells, and dendritic cells like (DC-like) cells in the necrotic intestinal epithelium. These findings suggest that vAh rapidly proliferated and spread through the catfish organs following bath challenge.

## Introduction

*Aeromonas hydrophila* is a Gram-negative, facultative anaerobic, motile bacterium that is the causative agent of motile *Aeromonas* septicemia (MAS) in fish. This bacterium is widely distributed in aquaculture and can cause significant losses in the presence of predisposing stressor (Plumb and Hanson, 2010). The disease in fish has two forms: acute hemorrhagic septicemia characterized by generalized edema, hemorrhage, and diffuse necrosis; and chronic ulcerative syndrome marked by the formation of deep dermal ulcers (Huizinga et al., 1979; Cipriano et al., 1984). The pathogenic potential of *A. hydrophila* has been related to several extracellular proteins and several virulence factors including hemolysins, cytotoxins, enterotoxins, proteases, lipases, leucocidins, endotoxin, surface polysaccharides (capsule, lipopolysaccharide, and glucan), iron-binding systems, exotoxins, extracellular enzymes, secretion systems, fimbriae and other non-filamentous adhesins, motility, and flagella (Daskalov, 2006).

Channel catfish (*Ictalurus punctatus*) is the most important cultured fish in the United States ($361 million total sales in 2015), and the majority of catfish production takes place in Mississippi ($191 million total sales in 2015) (United States Department of Agriculture [USDA], 2016). Since 2009, a highly virulent *A. hydrophila* strain (vAh) has caused severe acute mortality in cultured catfish in Alabama, Mississippi, and Arkansas (Hemstreet, 2010; Da Silva et al., 2012). The disease impacts primarily food-sized, marketable fish in summer months when water temperature reaches up to 35°C in catfish ponds (Zhang X. et al., 2014). The disease has cost about three million pounds of food-size catfish annually (Hemstreet, 2010). These epizootics have continued to affect catfish production adversely (Bebak et al., 2015).

There have been a few histopathological studies of *A. hydrophila* in catfish (Bach et al., 1978; Grizzle and Kiryu, 1993; Alagappan et al., 2009; Islam et al., 2013). These studies have focused mostly on clinical signs and description of internal lesions after experimental injection of fish. Recently, cutaneous lesions and widespread hyperemia of abdominal organs, as well as petechial and ecchymotic hemorrhages scattered over mesenteric tissues, were observed by light microscopy (LM) in catfish infected with vAh by immersion (Rasmussen-Ivey et al., 2016). Here we present, for the first time, sequential in-depth characterization of the histopathological and ultrastructural changes in channel catfish in a period of 2 days after bath challenge.

## Materials and Methods

### Bacterial Culture and Catfish Challenges

*Aeromonas hydrophila* strain ML09-119 (vAh type strain) isolated from diseased catfish during a MAS pond outbreak was used in this study (Griffin et al., 2013). The strain was grown on brain heart infusion (BHI) agar or broth (Difco, Sparks, MD, United States) and incubated at 37°C (Janda and Abbott, 2010).

Fish experiments were conducted by following a protocol approved by the Institutional Animal Care and Use Committee at Mississippi State University. Specific pathogen free (SPF) channel catfish (120 catfish, 64.4 ± 5.8 g and 21.5 ± 3.2 cm) were obtained from the College’s SPF fish hatchery and stocked in 12 40-L tanks (10 catfish/tank) with a continuous water flow and aeration. Water temperature was kept at 32°C and catfish were fed twice a day. Chlorine, dissolved oxygen, and temperature of the tanks were monitored daily. After 1 week of acclimation, tanks were randomly assigned to three groups (mortality, pathology, and control), and each group contained four tanks. The mortality and pathology groups were challenged by vAh culture, and used for determination of fish mortality calculation and sequential pathology analysis, respectively. The control group (BHI exposed) served as a negative control for both mortality and pathology groups.

Bacterial bath challenge was conducted as described previously (Abdelhamed et al., 2016). Briefly, the water level in each tank was dropped to 10 l, and 100 ml of overnight culture was added to each tank to yield approximately 4.3 × 10^10^ CFU/ml water. After 6 h at 32°C with aeration, water flow was restored to allow dilution and elimination of vAh. The bacterial concentration in bath challenge was calculated by determining the CFU number of the original culture (serial dilutions, plating, and colony counting). Fish mortalities in mortality group were checked every day for 2 weeks. To confirm the vAh as the cause of mortality, swabs from spleen and anterior kidney of dead fish were cultured on BHI agar.

After calculation of the percent mortalities, arcsine transformed percent mortality values from the mortality and control groups were compared by Student’s *t*-test.

### Histopathological Examination of Catfish Tissues

Six catfish were sampled randomly from the pathology group and 2 catfish were sampled randomly from the control group at 1, 3, 5, 6, 24, and 48 h post-challenge (HPC) for pathological analysis. After euthanatization by tricaine methanesulfonate overdose (300 mg/l), the whole fish were fixed in 10% neutral buffered formalin for at least 24 h, decalcified in Kristensen’s fluid, neutralized, and serially cross-sectioned. The fish tissue sections were placed in plastic cassettes and were processed (gradual dehydration in 70–100% alcohol, clearing in xylene, and paraffin wax embedding). Five micron thick sections were cut on a microtome (Lecia RM 2255), then stained with hematoxylin and eosin (H&E) (Lecia ST 5020, Lecia CV 5030) or by standard Giemsa staining. Slides were examined with a light microscope (Olympus BX43 with DP72 digital camera and CellSens software package).

### Ultrastructural Examination of Catfish Tissues

Tissue specimens from spleen, stomach, intestine, gills, and kidney from six fish were collected at 48 HPC and fixed in 0.5 Karnovesky’s solution in 0.1 M Na cacodylate buffer and then post-fixed in 1% osmic tetroxide. Tissues were then dehydrated in the serial concentration of alcohol, acetone, and embedded in resin. Thick sections (0.5 microns) were cut using ultramicrotome, stained with toluidine blue, and examined using LM. The ultrathin sections (80 nm) of selected areas were stained with uranyl acetate and lead citrate. The stained sections were examined and photographed using transmission electron microscopy (TEM) (Jeol TEM-1230).

## Results

### Catfish Mortality after vAh Challange

Fish mortality in the vAh challenge group was significantly higher than that of control group (*P* < 0.05; 62.4% vs. 0%), and most of the mortalities occurred within 5–48 HPC. We observed pure colonies of *A. hydrophila* strain on BHI agar cultured from spleen and anterior kidney of dead fish. The control fish yielded no mortality.

### Histopathological Findings

Six catfish were examined for histopathological changes at 1, 3, 5, 6, 24, and 48 HPC. Three fish out of six showed lesion in spleen and stomach at 1 and 3 HPC without any detectable lesions in the other organs. After 3 h and until 48 h spleen, stomach, and intestine showed exaggerated lesions in the majority of examined catfish. Multifocal lesions were detected in liver, kidney, and gills at 24 and 48 HPC.

#### Spleen

**FIGURE 1:**
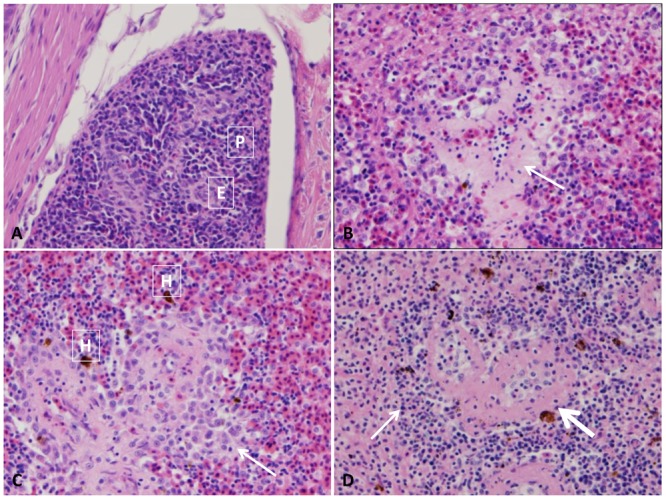
Photomicrograph of control and infected catfish spleen. **(A)** Photomicrograph of control spleen showed normal architecture with ellipsoids (E) beside mixed red and white pulp (P). **(B)** Infected spleen showed mild splenitis with an accumulation of proteinaceous substance around ellipsoid arterioles (arrow) at 5 HPC, 100×. **(C)** Moderate splenitis with numerous macrophages and lymphocytes aggregated around ellipsoids (arrow) in addition to hemosiderosis (H) at 24 HPC, 400×. **(D)** Severe splenitis with diffuse fibrinoid necrosis of ellipsoidal sheath (thick arrow) and aggregation of inflammatory cells (thin arrow) at 48 HPC, 400×, H&E.

The splenic section of control catfish revealed their normal red pulp structure with normally compacted ellipsoids (**Figure 1A**). At 1–3 HPC, splenomegaly with a massive expansion of red pulp by many erythrocytes and ellipsoidal compression of white pulp were detected in three fish. At 5 HPC, approximately 95–100% of spleen showed expansion of the ellipsoid sheaths 2–3 times than normal by a light pink hyaline material with erythrocyte fragments and transmigratory leukocytes in addition to the expansion of red pulp by erythrocytes (**Figure 1B**). Later at 24 HPC, ellipsoid sheaths were surrounded by 2–3 layers of macrophages, erythrocyte fragments, and karyorrhectic debris. Macrophages within these aggregates contain large, irregular, brown granular pigments (likely hemosiderin) in the six examined fish (100%) (**Figure 1C**). While at 48 HPC, fibrinous splenitis appeared with complete replacement of ellipsoid sheaths by thick homogenous eosinophilic material admixed with karyorrhectic debris. These lesions were walled-off by 2 or 3 layers of macrophages and lymphocytes. Depletion of erythrocyte within red pulp, which is replaced by fibrinous material was evident in the whole fish (100%) (**Figure 1D**). Hemosiderosis were seen in the majority of examined fish at the different time points.

#### Stomach

**FIGURE 2:**
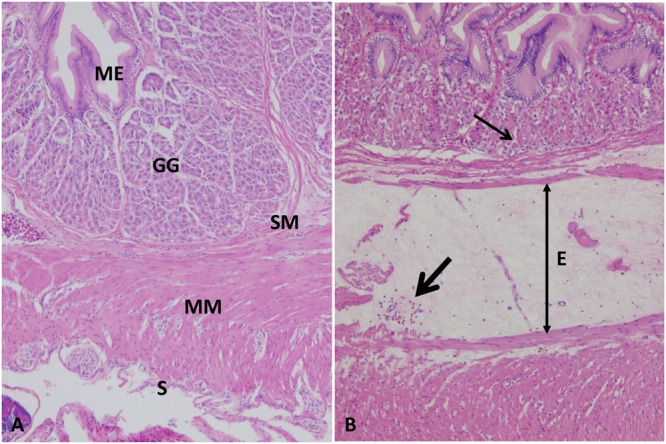
Photomicrograph of control and infected catfish stomach. **(A)** Control stomach showing its normal organization, mucosal epithelium (ME) with a gastric gland (GG) in its lamina propria, submucosa (SM), muscularis (MM) and serosa (S), 100×, H&E. **(B)** Infected stomach showed extensive edema (E) and hemorrhage (thick arrow) of submucosa with necrosis of its gastric glands (thin arrow), 100×, H&E.

Normal arrangement of gastric layers appeared in the stomach of the control group including mucosa, lamina propria with the gastric gland, muscularis, and serosal layers (**Figure 2A**). At 1–3 HPC, mild to moderate edema were detected in three fish out of six. From 5 to 48 HPC, diffuse expansion of submucosa by 2- to 3-fold more than normal with pale eosinophilic proteinaceous fluid (edema) were observed in the entire stomach wall of each fish examined. Also, mild hemorrhage with few mononuclear cells was detected in the edematous fluid. Additionally, gastric gland cells were often necrotic surrounded with few lymphocytes and macrophages filled with ingested debris were often observed (**Figure 2B**).

#### Intestine

**FIGURE 3:**
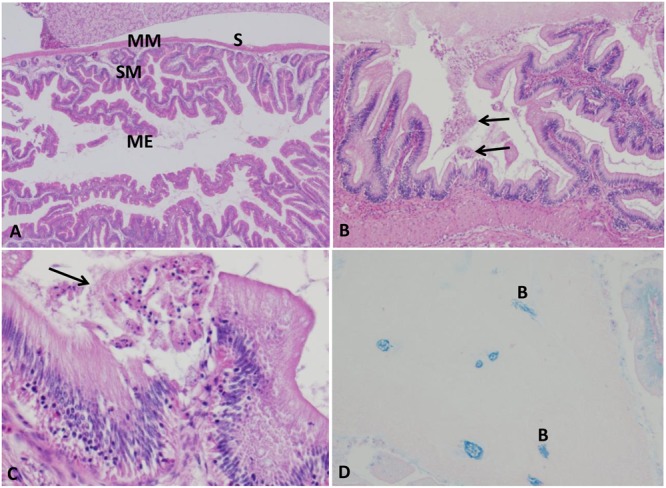
Histopathological changes in the catfish intestine. **(A)** Photomicrograph of control intestine showed its characteristic layers: mucosal epithelium (ME), submucosa (SM), muscularis (MM), and serosa (S). **(B,C)** Intestine of infected catfish showed epithelial necrosis with sloughed necrotic debris in its lumen (arrows) 100×, 400×. **(D)** Multifocal aggregation of bacteria in the intestinal lumen (B), 200×, Giemsa stain.

Intestine of control group exhibited normal intestinal layers including mucosa, submucosa, muscularis, and serosa (**Figure 3A**). At 5 HPC, approximately 75% of the surface of entire mucosal layer were necrotic in two fish. At 24 and 48 HPC, extensive necrosis of the surface of entire mucosa with an accumulation of necrotic enterocytes and homogenous substance within the lumen in six fish (**Figures 3B,C**). Multifocal clumps of monomorphic rod bacteria were detected in the intetstinal lumen (**Figure 3D**).

#### Gills

**FIGURE 4:**
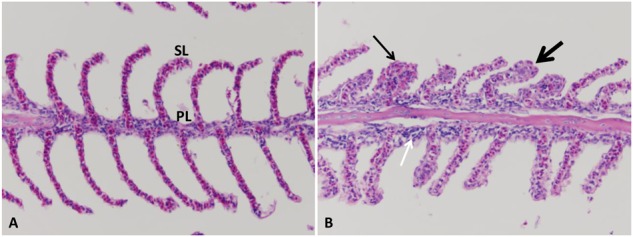
Photomicrograph of control and infected catfish gills. **(A)** Control gills showed normal primary lamellae (PL) and secondary lamellae (SL), 200×, H&E. **(B)** Infected gills showed thickening (thin arrow) and clubbing of the primary and secondary lamellae (thick arrow) with moderate numbers of lymphocytic infiltration (white arrow) at 24 HPC 200×, H&E.

Normal primary and secondary lamellae were seen in the control group (**Figure 4A**). At the first 1–3 HPC, no detectable lesions were observed in any of the examined catfish. From 5 to 6 HPC mild sloughed epithelial cells of secondary lamellae were seen in the majority of examined gills. At 24 and 48 HPC, thickening of both primary and secondary lamellae in approximately 75–80% of gill lamellar surface in addition to clubbing of the secondary and primary lamellae were observed. A moderate number of lymphocyte invaded the lamellae (**Figure 4B**).

#### Kidneys

**FIGURE 5:**
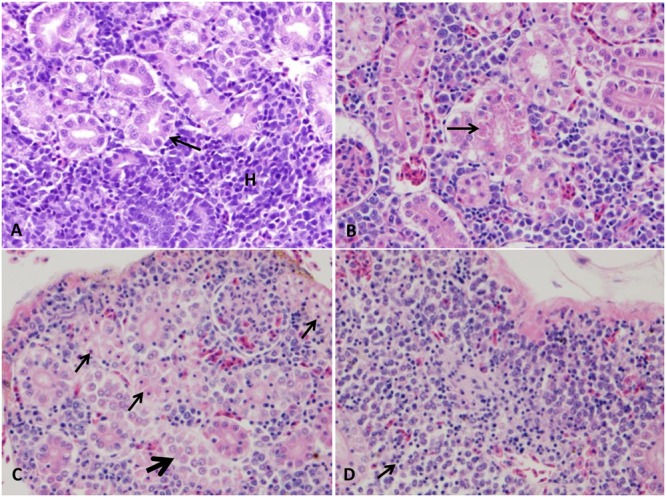
Photomicrograph of control and infected catfish posterior kidney. **(A)** Normal organization of posterior kidney with many renal tubules (arrow) and area of hematopoietic tissue in between (H). **(B)** Infected posterior kidney showing hyaline droplet accumulation in its tubular epithelium (arrow) at 24 HPC 400×, H&E. **(C)** Diffuse necrosis of renal tubule (thin arrow) beside separation of renal tubular epithelium from its basement membrane with karyolysis of its nucleus (thick arrow) at 48 HPC, 400×, H&E. **(D)** Severe interstitial nephritis replaced the renal tubules (arrow) at 48 HPC 400×, H&E.

**FIGURE 6:**
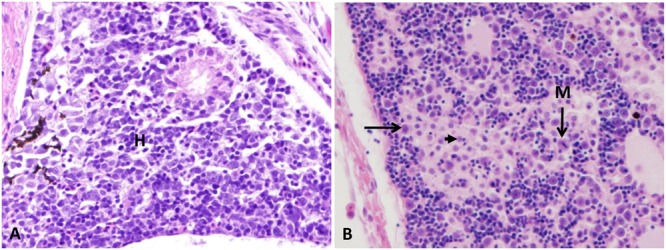
Photomicrograph control and infected anterior kidney. **(A)** Normal arrangement of the anterior kidney with normal hematopoietic aggregation (H). **(B)** Infected head kidney showing moderate pronephritis (left arrow) with some cells appear necrotic (arrowhead) and others showed mitotic figures (M) at 48 HPC 400×, H&E.

Posterior kidney (mesonephros) of control group exhibited normal renal tubules with normal hematopoietic tissue in between (**Figure 5A**). At 24 HPC, approximately 75% of the posterior kidney showed hyaline droplet accumulation in the renal tubular epithelium in the majority of examined fish (**Figure 5B**). At 48 HPC, interstitial nephritis with diffuse coagulative necrosis of tubular epithelial cells was observed in the posterior kidney in the majority of examined fish (**Figures 5C,D**). Anterior kidney (pronephros) showed the general organization of hematopoietic tissue with different cell types randomly aggregated in the control group (**Figure 6A**). At 48 HPC, approximately 25% of pronephros showed moderate multifocal pronephritis characterized by the aggregation of macrophages. Some cells appeared necrotic with pyknotic and karyorrhectic debris, while others showed mitotic figures within and around hematopoietic cells in four fish (**Figure 6B**).

#### Liver

**FIGURE 7:**
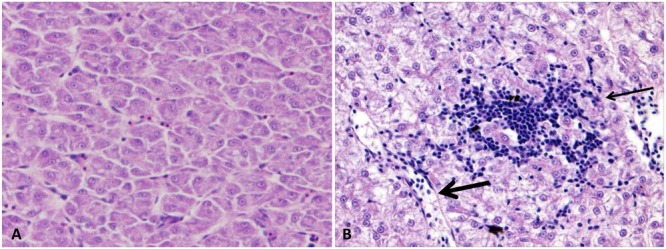
Photomicrograph of control and infected catfish liver. **(A)** The liver is showing the normal organization of polygonal hepatic cells and blood capillary. **(B)** Infected liver showed focal area of lymphocytic aggregation (thin arrow) migrate from dilated blood vessels (thick arrow) toward necrotic hepatic cells at 48 HPC, 400×, H&E.

Normal polygonal hepatocyte with normal blood vessels were seen in the control group (**Figure 7A**). No detectable lesions were observed in the liver at 1, 3, 5, 6, and 24 HPC. At 48 HPC, mild multifocal hepatitis in approximately 25% of hepatic parenchyma with focal lymphocytic aggregation around dilated blood vessels was appeared in three fish (**Figure 7B**).

### Ultrastructural Findings

Ultrastructural changes were reported in the internal organs of six catfish at 48 HPC. Although most of them showed ultrastructural lesions, three showed diffusion of bacterial cells with the secretion of its outer membrane vesicles (OMVs) in all organs especially in the spleen and gills.

#### Spleen

**FIGURE 8:**
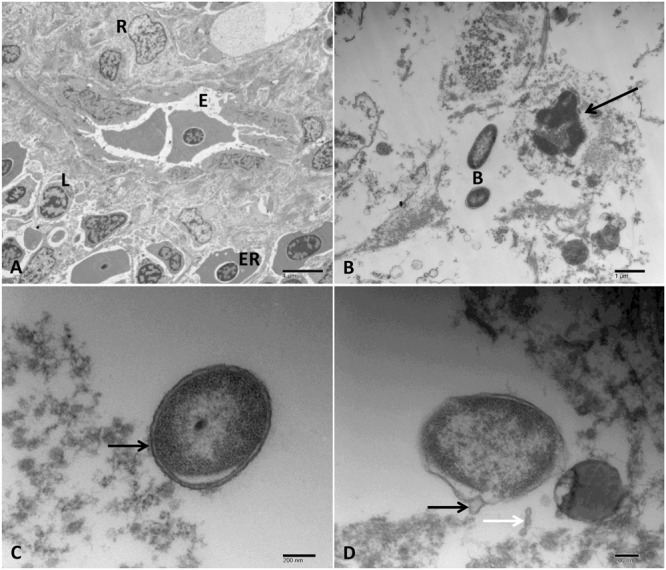
Transmission electron microscopy of control and infected spleen at 48 HPC. **(A)** Normal spleen with normal ellipsoids (E) surrounded by reticular cells (R) and mixed pulp and characterized by the presence of multiple erythrocytes (ER) and lymphocyte (L). **(B)** Infected spleen with the loss of its cellularity and complete lysis of the remaining cells. The cell nucleus had pyknosis with clumping of the chromatin (arrow) beside extracellular bacteria (B). **(C)** Outer membrane vesicles linked to the bacterial cell membrane (arrow). **(D)** Bulges around the bacterial envelope (black arrow) and chain of vesicles released near the site of its origination (white arrow). Bar = 4 μm **(A)**, 1 μm **(B)**, and 200 nm **(C,D)**.

Spleen exhibited normal architecture in control catfish with normal ellipsoids lined by cuboidal cells and surrounded by a layer of reticular cells and macrophages as well as red pulp sinuses filled with erythrocytes and surrounded by the different type of cells mainly lymphocytes (**Figure 8A**). The infected catfish spleen showed complete destruction of endothelial and reticular cells with loss of the cellularity, and the remaining cells showed pyknosis and cytolysis. An abundance of dividing bacteria were found extracellularly between splenic cellular debris (**Figure 8B**). Protrusion of OMVs from the bacterial outer membrane was observed. The OMVs were found either attached to the outer membrane or secreted nearby its origin as a vesicular chain in some cells (**Figures 8C,D**).

#### Stomach

**FIGURE 9:**
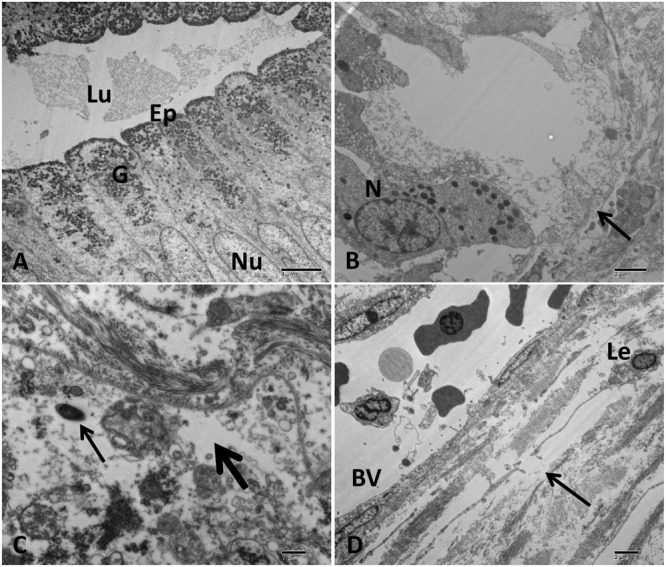
Transmission electron microscopy of control and infected catfish stomach at 48 HPC. **(A)** The normal structure of stomach with columnar epithelial cells (Ep), many granules concentrated on the apical part of epithelial cells (G), normal nucleus (Nu), and lumen contains secretion (Lu). **(B)** Severe damage to gastric gland with complete loss of its normal structure (arrow) beside neutrophilic infiltrations (N). **(C)** Extensive edematous fluid widely separated collagen bundles and necrosis of cellular structure of connective tissue (thick arrow) beside bacterial cell (thin arrow). **(D)** Edematous fluid (arrow) containing leukocyte (Le) accumulated around blood vessels (BV). Bar = 4 μm **(A)**, 2 μm **(B,D)** and 1 μm **(C)**.

The control group showed a normal gastric structure with columnar epithelial and mucous cells (**Figure 9A**). The challenged catfish stomach showed moderate to severe damage of gastric gland plasma membrane with nuclear chromatin condensation and cytoplasmic organelles lysis. Additionally, infiltration of neutrophils and lymphocytes around necrotic gastric gland were observed (**Figure 9B**). Edematous fluid with necrotic debris was noted in the interstitial submucosa and around blood vessels. A few number of bacterial cells and lymphocytes were found free in the edematous fluid (**Figures 9C,D**).

#### Intestine

**FIGURE 10:**
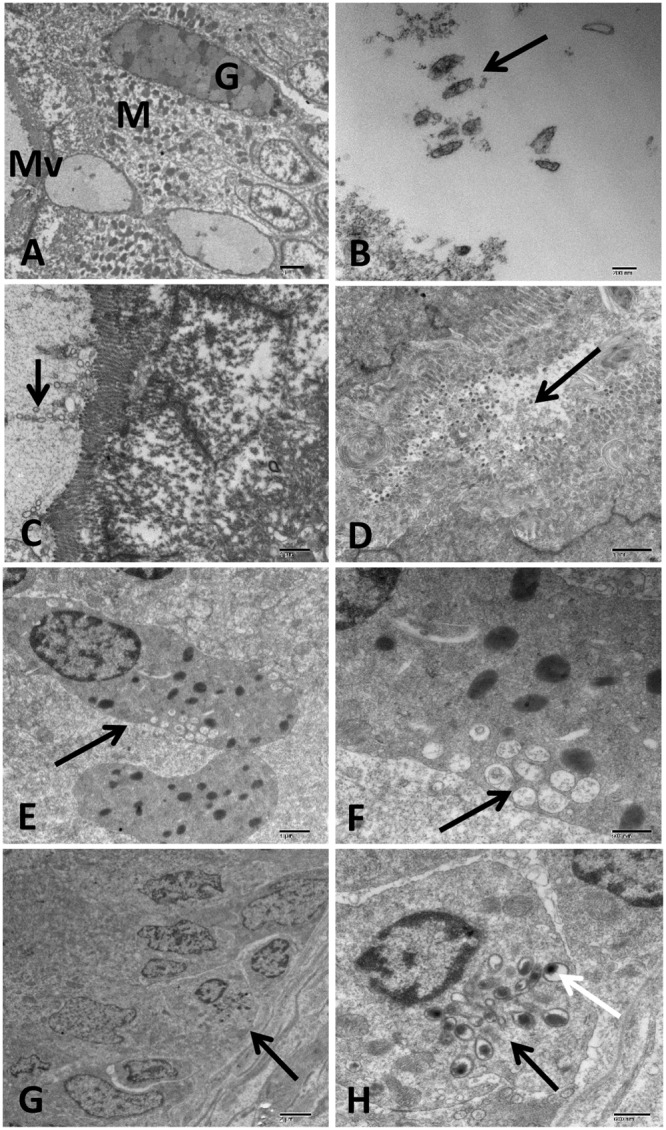
Transmission electron microscopy of control and infected catfish intestine at 48 HPC. **(A)** Intestine of control fish with normal microvilli (MV) tightly packed to enterocyte surface, numerous goblet cells (G) and enterocyte with several spherical mitochondria (M) and normal nucleus scattered inside the enterocyte. **(B)** Infected intestine with many damaged bacterial cells inside its lumen (arrow). **(C)** Intestine of infected catfish showed up to four vesicular chains attached to intestinal microvillus with little change in enterocyte structure (arrow). **(D)** Severe enterocyte damage with the complete deterioration of its microvilli and cytoplasmic organelles beside detached enterocyte filled its lumen (arrow). **(E,F)** Degranulate eosinophilic granular cells (EGC) invaded the necrotic enterocyte characterized by electron-lucent multivesicular granules accumulated in its peripheral cytoplasm beside few number of electron-dense granules (arrow). **(G,H)** Dendritic cells like (DC-Like) invaded the intestinal epithelium (arrow) characterized by birbeck like granules (black arrow) associated with electron dense lysosome and phagocytic particles (white arrow). Bar = 2 μm **(A,G)**, 200 nm **(B)**, 1 μm **(C–E)**, 500 nm **(F)** and 600 nm **(H)**.

Intestine of control catfish revealed normal enterocyte with several spherical mitochondria and scattered goblet cells. The luminal surface of enterocyte possessed closely packed parallel microvilli (**Figure 10A**). The infected group revealed the presence of many damaged bacterial cells inside intestinal lumen with up to four chains of OMVs attached to the luminal surface of enterocytes (**Figures 10B,C**). Severe damage of the enterocyte with the complete deterioration of its microvilli and organelles were observed (**Figure 10D**). The necrotic enterocytes were invaded by numerous defensive cells such as degranulate (activated) eosinophilic granular cells (EGC) and dendritic cell like (DC-Like) cells. The degranulated EGC had many electron lucent multivesicular granules accumulated in its peripheral cytoplasm beside few electron-dense crystalloids granules (**Figures 10E,F**). The DC-Like cells had birbeck-like granules associated with electron dense lysosomal structure and phagocytic particles were also observed (**Figures 10G,H**).

#### Gills

**FIGURE 11:**
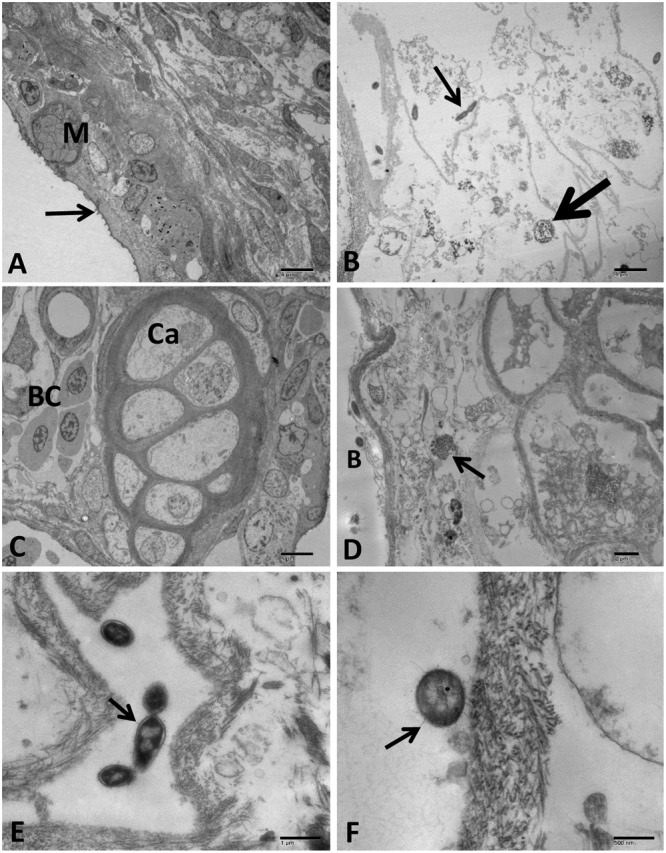
Transmission electron microscopy of control and infected catfish gills at 48 HPC. **(A)** Control gills showing normal primary lamellae epithelium with different cell types: mucous cells (M) and pavement cells with micro fridge (arrow). **(B)** Gills of the infected group showing diffuse divided bacteria (thin arrow) admixed with sloughed necrotic lamellar epithelial lining (thick arrow). **(C)** Control gills showing cartilaginous portion (Ca) and blood cells (BC) inside the primary lamellar structures. **(D)** Necrosis of interstitial connective tissue surrounding cartilaginous portion (arrow) beside a bacterial (B) attached to lamellar epithelium completely destructed. **(E)** Multiple dividing bacteria showing secretion of many OMV chains attached to its envelope (arrow). **(F)** Bacteria attached to the remnant tissue of gill lamellae with the secretion of OMVs (arrow). Bar = 4 μm **(A–D)**, 1 μm **(E)**, 500 and 200 nm **(F)**.

Control group showed normal gill mucous and pavement cells with microfidge in addition to chloride cells (**Figure 11A**). Normal gills chondrocyte structure surrounded by interstitial connective tissue were observed in control group (**Figure 11C**). In the challenged catfish, gills showed complete loss of the branchial lamellar epithelium beside diffuse replicated bacterial cells with many chains of OMVs attached and separated beside its origin (**Figures 11B,E,F**). Additionally, necrosis in the interstitial connective tissue and chondrocyte was seen in most of the examined fish (**Figure 11D**).

#### Anterior Kidney

**FIGURE 12:**
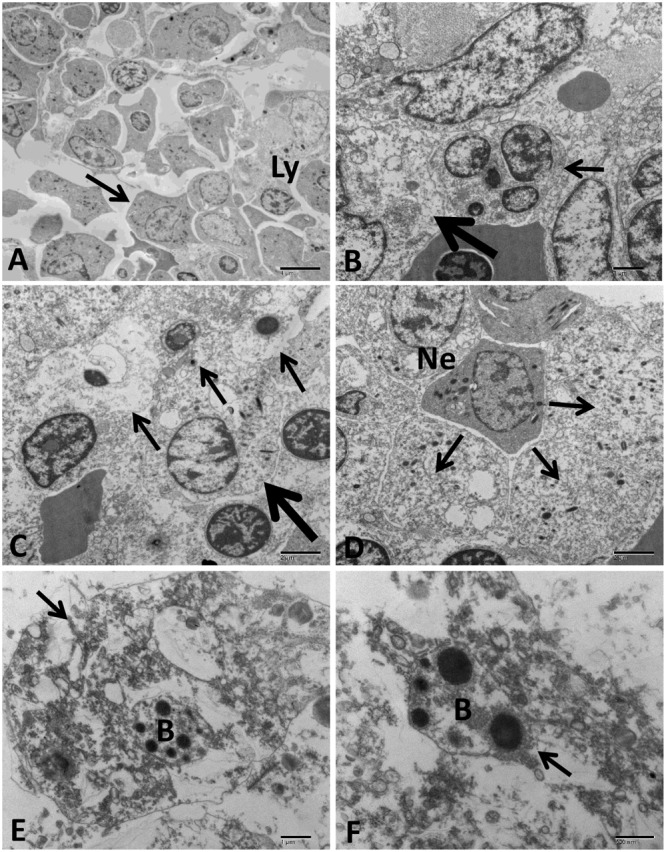
Transmission electron microscopy of control and infected catfish anterior kidney at 48 HPC. **(A)** The normal structure of anterior kidney with a different type of cells including lymphocyte (Ly), and granulocytic cells (arrow) in the hematopoietic tissue. **(B)** Infected anterior kidney showing degenerative cells with dilated cytoplasmic organelles (thick arrow) beside hyperplastic cells had mitotic figures (thin arrow). **(C)** Three necrotic cells with complete lysis of its cytoplasmic organelles and clumped chromatin of its nucleus (thin arrows) beside degenerative granulocytic cells showed mitochondrial dilation (thick arrow). **(D)** Severe necrosis of multiple granulocytic cell types (arrows) beside infiltrated neutrophil with its characteristic granules inside the cytoplasm (Ne). **(E)** Anterior kidney phagocytic cells showed disruption of its plasma membrane, cytoplasmic and nuclear structural damage (arrow) beside degenerated bacteria inside its phagosome (B). **(F)** Phagocytic cell with multiple degenerated bacteria (B) inside its phagosome (arrow) (B). Bar = 4 μm **(A)**, 2 μm **(C,D)**, 1 μm **(B,E)**, and 800 nm **(F)**.

Anterior kidney of the control group showed normal architecture with the lymphocyte, granulocyte, and endocrine aggregate regions (**Figure 12A**). The hematopoietic tissue of the infected group showed degenerative cells with dilated organelles, necrotic cells with complete loss of its cytoplasmic membrane besides cytoplasmic organelle lysis and condensed nuclear chromatin in addition to hyperplasia with mitotic figure and neutrophilic infiltration (**Figures 12B–D**). Phagocytic cells characterized by cytoplasmic membrane disruption, damage of cytoplasmic organelles, and nuclear structures. Also, many degenerated bacteria engulfed inside phagosomes of phagocytic cells were observed (**Figures 12E,F**).

## Discussion

The objective of this study was to determine histopathological and ultrastructural changes in catfish fingerlings after vAh challenge. Previous studies have employed intraperitoneal (IP) injection to determine the virulence of *A. hydrophila* isolates and to investigate the effect of prophylactic treatments on prevention of MAS (Pridgeon and Klesius, 2011; Zhang D. et al., 2014). In this work, we utilized bath challenge in catfish. Although injection method is more reproducible than immersion or bath method, it is incongruous with the natural infection because of the bypassed skin and mucous innate defenses (Bach et al., 1978; Walters and Plumb, 1980; Miyazaki and Kaige, 1985). The immersion or bath challenge mimics natural route of infection in aquatic conditions (Ventura and Grizzle, 1988).

The incubation period of MAS varies between 2 and 4 days in natural infections and 8–48 h in experimental infection models (Bach et al., 1978; Huizinga et al., 1979; Yardimci and Aydin, 2011; Anyanwu et al., 2015). In the current study, the pathological lesions were observed in spleen and stomach at 1 HPC, which is earlier than the range indicated for experimental infection. This might be due to higher virulence of the epizootic *A. hydrophila* strain ML09-119, catfish mortalities in ponds occurred within 12 h (Hemstreet, 2010).

The LM and TEM observations demonstrated that vAh cells rapidly gained entry into catfish organs and caused histopathological changes in spleen, stomach, intestine, gill, kidney and liver following the bath challenge. The lesions observed in this study are agree with the majority findings seen in natural or experimental *A. hydrophila* infections in other fish species including Nile tilapia (*Oreochromis niloticus*), crucian carp (*Carassius carassius*), iridescent shark (*Pangasius hypophthalmus*), and rainbow trout (*Oncorhynchus mykiss*) (Miyazaki and Kaige, 1985; Candan, 1990; Kumar et al., 2015).

The pathological lesions in the spleen were first evident by 1 HPC. This might be due to spleen being the primary initial target organ and/or the importance of the spleen in monitoring the blood stream against invading pathogen and its toxin. In a previous report, the channel catfish orally infected with *A. hydrophila* showed no or very little spleen abnormality, while, on the catfish infected intraperitoneally showed abnormal spleen in a short time and rapid onset of acute systemic infection (Bach et al., 1978).

Stomach exhibited a significant increase in the thickness of gastric submucosa by the edematous fluid with a focal area of hemorrhage. No previous study revealed the effect of *A. hydrophila* on the stomach except one study in Nile tilapia showed grossly hyperemia and congestion of stomach after infection with *A. hydrophila* (Yardimci and Aydin, 2011). This may be due to the bath challenge model; the previous study mainly used IP injection. The observations on intestine indicated necrosis of apical part of intestinal mucosa with little edema in the wall and accumulation of bacterial cells in intestinal lumen, which was similar to previous studies where extensive necrosis of the intestinal villi with extensive bacterial invasion was observed in crucian carp (Miyazaki and Kaige, 1985) and spotted snakehead (*Channa punctatus*) (Poline et al., 2014) following *A. hydrophila* infection.

Gills showed clubbing and thickening of lamellae with leukocytic infiltration. Severe branchial damage with many dividing *A. hydrophila* was observed. A previous study reported hyperplasia and leukocytic infiltration in gills of catfish with *A. hydrophila* infection (Laith and Najiah, 2013).

The anterior kidney showed mild multifocal inflammation characterized by large macrophages and karyorrhectic debris. The posterior kidney showed hyaline droplet accumulation in the renal tubular epithelium and interstitial nephritis with necrosis of tubular epithelial cells. In a previous study, diffuse necrosis was reported in the kidney of crucian carp (Miyazaki and Kaige, 1985), Nile tilapia (Yardimci and Aydin, 2011), and channel catfish (Ventura and Grizzle, 1988). Extensive hemorrhage and diffuse infiltration of mononuclear cells with marked edema in tubules of rohu (*Labeo Rohita*) fingerlings (Kumar, 2007). Liver tissue exhibited multifocal aggregation of inflammatory cells surrounded individual necrotic hepatocyte. A similar type of tissue destruction was reported in the walking catfish (*Claris batrachus*) (Angka, 1990), rainbow trout (*Salmo gairdneri*) (Candan, 1990), and channel catfish (*I. punctatus*) (Ventura and Grizzle, 1988) infected experimentally with *A. hydrophila.* The reason of the acute hemorrhage and necrosis of liver and kidney was reported to be associated with the release of toxins and extracellular products such as hemolysin, protease, elastase produced by *A. hydrophila* (Donta and Haddow, 1978; Asao et al., 1984; Lallier et al., 1984; Afifi et al., 2000), leading to rapid death due to organ failure (Huizinga et al., 1979).

One of the major pathological changes in the present study included hemosiderosis, especially in the spleen. The reason of the hemosiderosis was reported to be associated with β-hemolysin that cause hemolysis inside the fish body followed by deposition of hemosiderin (Miyazaki and Kaige, 1985).

The TEM examination of the spleen revealed complete lysis of its normal structure beside many bacterial cells diffused extracellularly in between the remnant of splenic tissue. A similar type of splenic lesion was reported in channel catfish following IP injection with *A. hydrophila* (Bach et al., 1978).

In the current study, extensive edema were accumulated around blood vessels of gastric gland with the presence of bacteria in it. The presence of this edematous fluid around the blood vessels confirm that the bacteria secrete toxins that damage the endothelial lining of the blood vessels and increase its permeability in the interstitial tissue. This is the first study reporting ultrastructural changes in the stomach following *A. hydrophila* infection in fish.

Ultrastructural changes in the intestinal tissue exhibited many degenerated bacteria inside the gut lumen with up to four chains of OMVs attached to the intestinal epithelium. This attachment was associated with deterioration of enterocytes and its microvilli with an accumulation of sloughed epithelium in the lumen. Infiltration of degranulate EGC and DC-Like was observed in the necrotic intestinal epithelium. The present paper reports for the first time activated EGC following *A. hydrophila* infection in the channel catfish. In a prior study, degranulation of EGC was reported in *Oncorhynchus mykiss* intestinal tissue injected with the extracellular product of *A. salmonicida* and *Vibrio anguillarum* (Vallejo and Ellis, 1989). In another study, activated eosinophils and monocyte were the main cells observed in mouse small intestinal tissue infected with *Aeromonas* spp. (Longa-Briceño et al., 2008). In addition to the presence of EGC, DC-Like cells were observed with its characteristic bribeck like granule associated with electron dense lysosomal structure, and phagocytic particles invaded the intestinal epithelium. The presence of lysosomal structure inside the bribeck-like granule of dendritic cells is evident for the antigen activation of these cells (Bucana et al., 1992). No previous study reported DC-Like cells in fish following *A. hydrophila* infection.

The most prominent effect in the hematopoietic tissue was increased number of necrotic phagocytic cells with many degenerated bacteria inside its phagolysosome. Similar phagocytic cells were reported in the renal hematopoietic tissue of spotted murrel (*Channa punctatus*) experimentally infected with *A. hydrophila* and *A. salmonicida* (Ghosh and Homechaudhuri, 2012). Furthermore, increase the number of phagocytic cells was reported in head kidney of Atlantic Salmon (*S. salar*) experimentally infected with *V. salmonicida* (Brattgjerd and Evensen, 1996).

The OMVs have been shown to play a major role in the transport and release of cytotoxic toxins and host tissue destruction in a significant number of pathogenic bacteria (Bergman et al., 2005; Parker et al., 2010). The TEM results demonstrated production of the OMVs and these vesicles are a part of the pathogenic mechanisms of vAh inducing severe damage to catfish internal organs. These findings agree with many authors study the formation and role of OMVs in the pathogenesis of *A. hydrophila* and other Gram-negative bacteria (Longa-Briceño et al., 2008; Andrea et al., 2015).

## Conclusion

Our data is the first record of histopathological alterations in catfish following bath challenge with vAh. Based on these results, it is reasonable to presume that the bath challenge can effectively mimic natural route of infections to reproduce MAS. The application of bath challenge successfully is expected to facilitate further studies including vaccine, antimicrobial testing, and probiotics applications for prevention of the disease.

## Author Contributions

HA, II, WB, ML, and AK conceived and designed the experiments. HA, II, and WB performed the experiments and analyzed the data. ML and AK contributed financial assistance. HA, II, WB, ML, and AK wrote the paper.

## Conflict of Interest Statement

The authors declare that the research was conducted in the absence of any commercial or financial relationships that could be construed as a potential conflict of interest.
